# Structural Racism, Health Inequities, and the Two-Edged Sword of Data: Structural Problems Require Structural Solutions

**DOI:** 10.3389/fpubh.2021.655447

**Published:** 2021-04-15

**Authors:** Nancy Krieger

**Affiliations:** Department of Social and Behavioral Sciences, Harvard T. H. Chan School of Public Health, Boston, MA, United States

**Keywords:** anti-racism, data governance, ecosocial theory of disease distribution, health equity, population health, politics of public health data, public health monitoring, structural racism

## Abstract

Analyzing the myriad ways in which structural racism systemically generates health inequities requires engaging with the profound challenges of conceptualizing, operationalizing, and analyzing the very data deployed—i. e., racialized categories—to document racialized health inequities. This essay, written in the aftermath of the January 6, 2021 vigilante anti-democratic white supremacist assault on the US Capitol, calls attention to the two-edged sword of data at play, reflecting long histories of support for and opposition to white supremacy and scientific racism. As illustrated by both past and present examples, including COVID-19, at issue are both the non-use (*Edge #1*) and problematic use (*Edge #2*) of data on racialized groups. Recognizing that structural problems require structural solutions, in this essay I propose a new two-part institutional mandate regarding the reporting and analysis of publicly-funded work involving racialized groups and health data and documentation as to why the proposed mandates are feasible. *Proposal/part 1* is to implement enforceable requirements that all US health data sets and research projects supported by government funds *must explicitly explain and justify their conceptualization of racialized groups and the metrics used to categorize them*. *Proposal/part 2* is that any individual-level health data by membership in racialized groups *must also be analyzed in relation to relevant data about racialized societal inequities*. A new opportunity arises as US government agencies re-engage with their work, out of the shadow of white grievance politics cast by the Trump Administration, to move forward with this structural proposal to aid the work for health equity.

## Introduction

Analyzing the myriad ways in which structural racism systemically generates health inequities (1–7)—that is, differences in health status across social groups that are unjust, avoidable, and in principle preventable (8–10)—requires scientific theory, hypotheses, data, and methods. This is standard science (11–13). What could be more obvious?

But when it comes to racialized health inequities, what appears obvious is rarely simple. Any attempt to analyze empirically—and provide evidence to alter—the causal processes by which structural racism produces health inequities, including by shaping discriminatory practices and policies of institutions and actions of individuals—must engage with the profound challenges of conceptualizing, operationalizing, and analyzing the very data deployed—i.e., racialized categories—to document racialized health inequities (1, 14–18).

**Figure 1:**
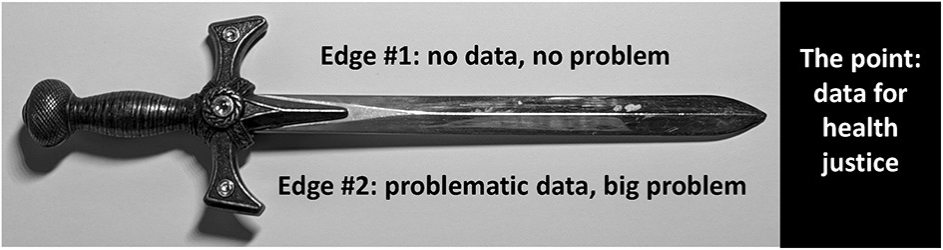
Structural injustice and the two-edged sword of data: (1) *Edge #1*: preventing documentation that injustice exists, (2) *Edge #2*: using problematic data in harmful ways that further entrench justice, vs. (3) looking instead to the point: data for health justice. [Note: the author created the figure and took the photo used; NO copyright issues are involved].

In this brief perspective, I accordingly call attention to the two-edged sword of data when it comes to racial justice and health (Figure 1). At issue are both the non-use (*Edge #1*) and problematic use (*Edge #2*) of data on racialized groups. To avoid being cut by either edge, my proposal – informed by the ecosocial theory of disease distribution and its constructs of embodying injustice along with accountability and agency for documenting and analyzing this causal process (1, 11, 17–20)—is to recognize that structural problems require structural solutions, including for racialized data.

Underscoring the urgency of these issues is the context in which I have prepared this essay. I began writing on January 8, 2021, 2 days after the flagrant violent assault on the US Capitol led by vigilante anti-democratic white supremacist, white nationalist (including white Christian nationalist), alt-right, and neo-Nazi groups, who sought to thwart a fair election and the peaceful transition of Presidential power (21–24). Unifying these groups is a belief in essentialist notion of “race,” a fear what they call “demographic replacement” (i.e., becoming white “minorities” in a multi-ethnic/racial society), and the politics of white grievance (whereby any attempts to name or limit white privilege are deemed anti-white racism) (25–30). More mainstream enablers have been seeking to cement conservative white minority rule, using the strategies of voter suppression and gerrymandering, while preserving the veneer of democratic governance (31–34). Also germane are wealthy supporters of an extreme “free market” political agenda and philosophy that holds government exists solely to defend private property—and jointly oppose government regulations (including to protect the public's health, e.g., protect against pollution, climate change, and environmental racism) and taxes to support government programs (especially if to rectify racial and economic injustice) (2, 33–38).

In such a context, how can an anti-racist science for health equity employ data on racialized groups? This is not a new question (15, 16, 30, 39–45)—indeed, in the US, these issues have been posed and debated in medical and public health literature for over 300 years (16, 39–45).

## Data Never Speak for Themselves

A crucial first point is that, contrary to its etymology, “data” is never a “given” (11, 16). Despite being the past participle of the Latin verb “dare,” i.e., “to give” (46), data instead are always produced by people, out of what they observe, fail to see, or suppress in the world in which they live (11–13, 47, 48). A corollary, in the case of people, is that a hallmark of privilege is who and what one can afford to ignore (49, 50). Translated to the realm of science, this means it is imperative to ask: who produces and controls the data? To what end? And engaging with what history? (11–13, 47–50).

Within the US, histories of the contested production and use of racialized data extend back to its origins as slave republic and settler-colonial nation (40, 43–45, 51–53). In the eighteenth century CE, racialized data were primarily produced and used to entrench injustice by the enfranchised minority of white men with property (52–54). These data characterized who was enslaved vs. free, and which Indigenous tribes and nations were vs. were not under colonial and then federal jurisdiction (51–54). Indeed, these data underpinned the first-ever decennial census in 1790, the first-ever census to be constitutionally mandated by a government anywhere (51–53), and which was designed to apportion political representation, allocate resources, and power, deeply marked by infamous 3/5 compromise that allowed slave-holding states partial counts of their enslaved but disenfranchised populations (52, 53). In this context, dominant white physicians held that racialized differences in health status, including the poorer health of enslaved persons and decimation of Indigenous peoples by colonial diseases, reflected the natural order of the world (6, 39, 40, 43–45, 55).

In the nineteenth century CE, the rise of the abolition movement, both Black- and white-led (56, 57), triggered a shift in use of racialized data (40, 43, 45). Thus, these abolitionists—including the first generation of credentialed African American physicians—began using racialized data, including on health status, to challenge the abominations of slavery, while supporters of slavery—including many physicians and scientists who embraced scientific racism—sought to use these same data to uphold the doctrine of white supremacy (40, 43, 45, 55–62). Then as now the debates turned on whether the racialized categories were constructed by people, to justify injustice, vs. so-called “natural” categories, reflecting innate or *a priori* differences; for health, the crux of the argument was whether racism vs. “race” accounted for observed differences in health status across racialized groups (30, 40, 43–45, 58–62).

Then as now key debates also concerned what additional data were required, beyond “race,” to contextualize the racialized health data. For example, Dr. James McCune Smith (1813–1865), the first credentialed Black American physician in the US (who received his medical degree in Scotland since no US medical school would admit Black students), in 1859 famously compared the similarly high prevalence of rickets among the enslaved Black population in the US South to destitute peasants in Ireland (45, 59, 60). By contrast, Dr. Samuel Cartwright (1793–1863), a prolific white pro-slavery physician, authored tract after tract about the biological “peculiarities” of Black persons that rendered them fit only to be slaves, without ever including any economic data (43–45, 62). Exemplifying the political salience of scientific racism was the inclusion of an essay by Cartwright (63) in the first print edition of the infamous US Supreme Court 1857 Dred Scott decision, which declared that Black Americans “had no rights which the white man was bound to respect …” (64–66).

In early the twentieth century CE, the same sorts of schisms existed, updated in relation to challenging vs. upholding Jim Crow, a multifaceted regime of legalized racial segregation, upheld by terror, and imposed in the 1880s, fueled by white Southern backlash to losing the Civil War and their enslaved workforce, plus opposition to civil rights gains won during Reconstruction (67–69). Kindred debates occurred over eugenics, with the latter strongly upheld by the US Supreme Court and leading scientists, University presidents, and more, who ushered in the passage of eugenic sterilization laws in 32 US states and also the eugenic-fostered federal Immigration Restriction Acts of 1924 and 1927 (30, 70–74).

Subsequently, during the 1950s and 1960s, racial/ethnic data featured prominently in fights for vs. against civil rights and for vs. against overturning the eugenic-era immigration restrictions (14, 30, 70–76). In the wake of major federal legislation passed in 1964 and 1965 which afforded new protection of civil rights and expanded immigration, official government racial/ethnic data was widely used to provide evidence of injustice, as opposed to justifying it (14, 51, 77–79). However, the successful civil rights strategy of using racialized data to demonstrate the existence of—and support alleviation of—what was termed “statistical discrimination” (or “disparate impact”) not surprisingly sparked conservative backlash, leading to two types of resistance (26, 32–34, 78–81).

- One was to try to suppress use of racial/ethnic data, thereby removing any evidence of harm requiring redress, as per the unsuccessful campaign of Proposition 54 in California in 2003 (82, 83). Deceptively dubbed the “Racial Privacy Initiative,” it sought to prohibit the state from recording or using any racial or ethnic data—and was defeated in part due to public health concerns about harms caused by concealment of data (83–87).
- The other approach was to require evidence of motivation, not just disparate impact (78, 79). The latest example is the outgoing Trump Administration's bid to require the Department of Justice to “narrowly enforce” Title VI of the Civil Rights Act, i.e., only “in cases where it could prove intentional discrimination, but no longer in instances where a policy or practice at issue had a “disparate impact” on minority or other groups” (88).

Concomitantly, groups concerned about racial justice have repeatedly expressed their concerns about how racialized data have repeatedly been deployed by those with power to stereotype and victim-blame, blaming allegedly “innate” biology and “chosen” cultures for health woes, starting with infant mortality and extending across the lifecourse, and newly including COVID-19 (1–7, 40, 42, 44, 89–92). The tension is real: both use and non-use of racialized data can wreak woes.

## The Two-Edged Sword of Data in Action: the Case of COVID-19 in the us

The problem of racialized health inequities and the two-edged sword of data is not unique to the US. Similar issues arise in other countries home to social inequities involving racialized groups, including but not limited to France, Portugal, Brazil, Mexico, the UK, Australia, New Zealand, and Canada—all countries whose histories are in differing ways bound up with legacies of colonialism, settler-colonialism, and slavery (15, 93–95). That said, because I am a US person, I offer an example of the two-edged sword in action in my country, in relation to a current calamity: COVID-19.

It is way too early to know how reporting of COVID-19 data by the US Centers for Disease Control and Prevention (CDC), including in relation to racialized groups, has been affected by political interference by the Trump administration, given emerging evidence of politically-motivated data suppression and distortion (96–99). That said, both edges of the sword were—and are still—in full view.

Early on in the pandemic, government data on COVID-19 data by racialized group was missing in action (100–103), despite federal health agencies routinely including racial/ethnic data for just about every disease and mortality outcome (16, 103–106). Instead, tallies and accounts of the burden of COVID-19 among racialized groups came mainly from investigative journalism and web-based data trackers created on the fly (107–116). In early June, propelled by the advocacy of racial justice activists who wanted these data to raise awareness and obtain resources for prevention for their communities, new Congressional legislation mandated the inclusion and reporting of COVID-19 data by race/ethnicity, to be fully implemented by no later than August 1, 2020 (112, 117, 118). This requirement, however, had no teeth: my team documented that between August 28 and September 16, 2020 fully 43% of the new COVID-19 cases added to the CDC roster were still missing racial/ethnic data (119). Welcome to *Edge #1* of the sword.

*Edge #2* cut when CDC reported what limited data it had stratified by race/ethnicity. Their focus initially was on *counts* (rather than rates) and concerned differences in the racial/ethnic composition of COVID-19 deaths vs. the total population (120). At the outset, the data for the deaths were on one website, and the data for the total population were on another, making it difficult to discern if the proportions differed (120). Worse, closer inspection of these data revealed a curious finding: contrary to reports coming from the field, the CDC's data in May 2020 indicated that white non-Hispanics were overrepresented, and Black Americans underrepresented, among COVID-19 deaths. Several of us worked to unravel this puzzle, and we soon determined the CDC had committed a classic Type III error: right answer to the wrong question (121). In brief, the CDC weighted the denominators for the US counties by the percent of total COVID-19 deaths occurring in that county within the state (120). Given who was hardest hit by COVID-19, the net effect was to deflate the denominators for the white non-Hispanic population and inflate the denominators for the other populations of color, thus inflating risk for the former and deflating risk for the latter (121). Why did the CDC do this? The stated reason was that they were concerned that the racial/ethnic composition of the places initially hit hard by COVID-19, especially NYC, differed from that of areas hit less hard—and their weighting procedure sought to “correct” this problem (120).

But: to ask and answer the question: how does racial/ethnic risk for COVID-19 mortality ***vary apart*** from how racial segregation affects who lives where is to ask and answer the entirely wrong question. By treating place and the lived experiences and impacts of residential segregation as nuisance factors, to be “corrected” for by weighting, the CDC reached the entirely wrong conclusion (121). It will be a task for future historians to establish the decisions, and likely politics, influencing the CDC's approach to data presentation on COVID-19 and racialized groups.

## Structural Racism, Data Needs, and Data Governance: Structural Solutions to Structural Problems

What then about the point of the sword: data for health equity? My structural suggestion: a two-part proposal to up the ante and create institutional mandates regarding publicly-supported work with racialized health data—whether public health monitoring, grant applications, or publications. The proposed steps are directly informed by ecosocial theory's emphasis on being explicit about agency and accountability at multiple levels, including both institutional and individual (17–20).

*Proposal/part 1* is to implement enforceable requirements that all US health data sets and research projects supported by government funds *must explicitly explain and justify their conceptualization of racialized groups and the metrics used to categorize them*. As shown in Table 1, there is precedent, admittedly weak, but a basis on which to build. Thus, since 1994 the National Institutes of Health (NIH) has required—with little enforcement—that grants report on and justify the number of “women and minorities” included, with “minorities” delimited using the US Office of Management and Budget categories (122–126). Moreover, since 2014, the new NIH policy on “Sex as a Biological Variable” (SABV) requires that reviewers rate all NIH grants' explanation of their approach to including “sex” as biological variable (127, 128)—albeit with no analogous requirements about how gender is conceptualized and analyzed (129). Numerous leading journals likewise proffer suggested—not mandatory—author guidelines regarding the use and interpretation of data on racialized groups (130, 131), albeit without analogous explicit instructions for reviewers (see Table 1). Hence, while none of these current institutional measures are sufficient, they do provide precedent for implementing structural steps to ensure that health agencies, organizations, and researchers must explicitly justify their conceptualization and analysis of racialized health data, and face consequences for not doing so.

**Table 1:** Structural solutions to the problems of structural racism and the two-edged sword of data so that it can point to health justice: proposed minimal data requirements for any health agencies, data systems, grant recipients, or journals receiving government support.

**Focus of structural rule**	**Structural requirement (minimal)**	**Examples suggesting feasibility of implementation–and limitations to be addressed**
Individual-level: membership in racialized group	1) Define how membership in the racialized group is conceptualized as a social variable and how it will be analyzed in relation to the individual-level socioeconomic measures and the community-level measures of structural racism 2) For purposes of comparability, and to enable calculation of population-based rates, minimally employ US census categories for “race” and “ethnicity,” which include Indigenous status, as stipulated by the federal Office of Management and Budget (OMB) categories (check all that apply) and required for all NIH grants that include human subjects (54, 126), and also categories for nativity (US born vs. foreign born) (147) or else birthplace as per US standard birth certificate (148)	1) Current journal requirements (regarding conceptualization of and justification for use of these data): International Committee of Medical Journal Editors [(130), p. 16–17]: “Because the relevance of such variables as age, sex, or ethnicity is not always known at the time of study design, researchers should aim for inclusion of representative populations into all study types and at a minimum provide descriptive data for these and other relevant demographic variables. Ensure correct use of the terms sex (when reporting biological factors) and gender (identity, psychosocial, or cultural factors), and, unless inappropriate, report the sex and/or gender of study participants, the sex of animals or cells, and describe the methods used to determine sex and gender. If the study was done involving an exclusive population, for example in only one sex, authors should justify why, except in obvious cases (e.g., prostate cancer). Authors should define how they determined race or ethnicity and justify their relevance. Authors should use neutral, precise, and respectful language to describe study participants and avoid the use of terminology that might stigmatize participants.” American Journal of Public Health (131): “If race/ethnicity is reported, the authors should indicate in the Methods section why race/ethnicity was assessed, how individuals were classified, what the classifications were, and whether the investigators or the participants selected the classifications.” *Limitations*: No requirement that reviewers evaluate submitted manuscripts in relation to these guidelines 2) National Institutes of Health (regarding requirements for including these data): (a) “Inclusion of Women and Minorities as Participants in Research Involving Human Subjects” and required enrollment tables in relation to federal categories of race, ethnicity, and sex (122–125) *Limitations*: no requirement that reviewers explicitly score grants in relation to approach taken to inclusion of racialized groups and how these groups are conceptualized and analyzed (b) “Consideration of Sex as a Biological Variable in NIH-funded Research,” which requires text in the Research Strategy section to “explain how relevant biological variables, such as sex, are factored into research designs and analyses for studies in vertebrate animals and humans,” with reviewers instructed to score grants in relation to what is stated (127, 128) *Limitation*: no requirements to address how gender identity and structurally embedded gender norms and institutional policies and practices are conceptualized or measured (129)
Individual-level measures of socioeconomic resources	1) Minimally employ US census categories for data on educational attainment (149): For persons age 25 and older: for self For persons under age 25: for parents or caregivers 2) Additional relevant US census measures pertaining to household income and number of persons (and age) supported by this income (to determine the poverty level), occupation, housing tenure, health insurance status, housing insecurity, food insecurity, etc. (149–151)	US standard birth certificates and death certificates: include data on educational attainment of parent(s) and educational attainment of the decedent, respectively (148) Routine collection of the additional proposed socioeconomic metrics in the US Census American Community Survey (149, 150) and the COVID-19 specific Household Pulse Survey (151)
Community-level measures of structural	1) Minimally use ZIP Code for residential address to link to ZIP Code Tabulation Area (ZCTA), but preferably geocode residential address to census tract level (132) 2) Minimally include US census compositional data on: median household income; poverty, and educational attainment (132, 133) 3) Minimally include metrics of social spatial polarization, including the Index of Concentration at the Extremes for economic segregation, racialized segregation, and racialized economic segregation (132), along with other measures of racial segregation (3–5) and data on historical redlining, if one of the cities or towns for which such maps were prepared for the US government in the 1930s (1, 3, 4, 152)	Proposed US census-derived metrics freely available at census tract and ZCTA level at: Public Health Disparities Geocoding Project (national coverage) (132) City Health Dashboard (available for over 750 US cities with populations more than 50,000) (133) Historical redlining data available for selected US cities at: “Not even past: social vulnerability and the legacy of redlining” (152)

But inclusion and reporting of racialized health data, as such, is only a first step. *Proposal/part 2* is that any individual-level health data by membership in racialized groups *must also be analyzed in relation to relevant data about racialized societal inequities*. As suggested in Table 1, this minimally means including both diverse metrics for socioeconomic position (at the individual- and community- levels) and exposure to structural racism (1–7, 18, 132–135). The latter can include explicit rule-based policies (e.g., involving voter suppression, or denial of Social Security benefits to domestic workers and agricultural workers) and also area-based or institutional measures that reflect racialized disparate impacts but not the rules *per se* (e.g., measures of racialized economic residential or occupational segregation, or racialized gaps in socioeconomic resources, incarceration rates, political representation, etc.) (1, 3, 135). Suggesting such steps are feasible, even in the midst of a fast-moving fearsome pandemic, scientific studies and data dashboards have generated striking evidence of the societal structuring of COVID-19 risks of exposure, illness, and death, using diverse metrics of residential segregation and racialized inequities in income, sick pay, and crowded housing (132, 136–143).

Granted, this two-part proposal for data justice is only one small step. Also needed is equity-oriented work on *data governance*, i.e., who has input into making the decisions about which data are required, informed by the tandem expertise of health equity researchers and other members of the communities whose data are at stake, affording the expertise of lived experience (2, 6, 47, 91, 92). Bringing a structural perspective to the data needs for data justice provides a start to the work at hand. Justifying implementation, continuing with the status quo and the harms produced is not acceptable.

## Final Reflections: Reckoning With Structural Racism and Data for Health Justice

On January 7, 2021, the day after the vigilante white supremacist anti-democratic assault on the US Capitol, the University College London (UCL) notably issued its first-ever institutional apology for its critical role in legitimizing the rise of eugenics and the horrors it has unleashed world-wide (144, 145). As prelude, the UCL last year stripped their buildings of the names of Francis Galton (1822–1911), who coined the term “eugenics,” and also his seminal field-building statistical disciples Karl Pearson (1857–1936) and Ronald Fisher (189–1962), who were, respectively, the first and second Professor of Eugenics at UCL (145, 146). The new apology minces no words, with the first two paragraphs stating (144):

*UCL acknowledges with deep regret that it played a fundamental role in the development, propagation and legitimization of eugenics. This dangerous ideology cemented the spurious idea that varieties of human life could be assigned different value. It provided justification for some of the most appalling crimes in human history: genocide, forced euthanasia, colonialism and other forms of mass murder and oppression based on racial and ableist hierarchy*.

*The legacies and consequences of eugenics still cause direct harm through the racism, antisemitism, ableism and other harmful stereotyping that they feed. These continue to impact on people's lives directly, driving discrimination and denying opportunity, access and representation*.

As this statement attests, the wounds cut by the two-edged sword of wrongly conceptualized and wrongly employed data on racialized groups not only have not healed—they continue to be cut anew.

A new opportunity arises as US government agencies re-engage with their work, out of the shadow of white grievance politics cast by the Trump Administration. It is time, long past time, to delineate new structural requirements for publicly-funded work using racialized health data, so that the point is clear: to expose the harmful impacts of structural racism on health and assess the beneficial impacts of anti-racist policies.

## Author Contributions

The author confirms being the sole contributor of this work and has approved it for publication.

## Conflict of Interest

The author declares that the research was conducted in the absence of any commercial or financial relationships that could be construed as a potential conflict of interest.
